# Supplementary material for: Machine Learning Accelerates Crystallization for Structure Determination
Source: Angew Chem Int Ed Engl. 2026 May 4;65(25):e1218503. doi: 10.1002/anie.1218503 (PMC13266958; doi:10.1002/anie.1218503)

checkCIF/PLATON report

Structure factors have been supplied for datablock(s) lcz010

THIS REPORT IS FOR GUIDANCE ONLY. IF USED AS PART OF A REVIEW PROCEDURE FOR PUBLICATION, IT SHOULD NOT REPLACE THE EXPERTISE OF AN EXPERIENCED CRYSTALLOGRAPHIC REFEREE.

No syntax errors found. CIF dictionary Interpreting this report

Datablock: lcz010

Bond precision:	C-C = 0.0148 A		Wavelength=1.54184
Cell:	a=21.1388(2)	b=21.1388(2)	c=12.6290(1)
	alpha=90	beta=90	gamma=120
Temperature:	100 K		

	Calculated	Reported
Volume	4887.20(10)	4887.20(10)
Space group	R 3 c	R 3 c
Hall group	R 3 -2"c	R 3 -2"c
Moiety formula	C15 H3 Ag3 F18 N6, C9 H12 O3	C5 H Ag F6 N2, C3 H4 O
Sum formula	C24 H15 Ag3 F18 N6 O3	C8 H5 Ag F6 N2 O
Mr	1101.03	367.01
Dx,g cm-3	2.245	2.245
Z	6	18
Mu (mm-1)	15.675	15.675
F000	3168.0	3168.0
F000’	3183.54	
h,k,lmax	26,26,16	23,23,16
Nref	2341[1177]	1908
Tmin,Tmax	0.113,0.111	0.638,1.000
Tmin’	0.014	

Correction method= # Reported T Limits: Tmin=0.638 Tmax=1.000
AbsCorr = MULTI-SCAN

```
Data completeness= 1.62/0.82        Theta(max)= 78.615

R(reflections)= 0.0344( 1903)       wR2(reflections)= 0.0939( 1908)

S = 1.087                  Npar= 164
```

The following ALERTS were generated. Each ALERT has the format
test-name_ALERT_alert-type_alert-level.
Click on the hyperlinks for more details of the test.

Alert level C

```
PLAT041_ALERT_1_C Calc. and Reported SumFormula    Strings  Differ     Please Check
              Calc: C24 H15 Ag3 F18 N6 O3
              Rep.: C8 H5 Ag F6 N2 O
PLAT042_ALERT_1_C Calc. and Reported MoietyFormula Strings  Differ     Please Check
              Calc: C15 H3 Ag3 F18 N6, C9 H12 O3
              Rep.: C5 H Ag F6 N2, C3 H4 O
PLAT090_ALERT_3_C Poor Data / Parameter Ratio (Zmax > 18) ........       7.05 Note
PLAT213_ALERT_2_C Atom F004           has ADP max/min Ratio .....        3.4 prolat
PLAT213_ALERT_2_C Atom F00E           has ADP max/min Ratio .....        3.8 prolat
PLAT213_ALERT_2_C Atom F00F           has ADP max/min Ratio .....        3.6 prolat
PLAT213_ALERT_2_C Atom F00K           has ADP max/min Ratio .....        3.3 prolat
PLAT250_ALERT_2_C Large U3/U1 Ratio for <U(i,j)> Tensor(Resd    1)       3.5 Note
PLAT342_ALERT_3_C Low Bond Precision on  C-C Bonds ...............   0.01483 Ang.
PLAT911_ALERT_3_C Missing FCF Refl Between Thmin & STh/L=    0.600         2 Report
                0  1  2,   0 10  2,
```

Alert level G

```
PLAT045_ALERT_1_G Calculated and Reported Z Differ by a Factor ...      0.333 Check
PLAT083_ALERT_2_G SHELXL Second Parameter in WGHT  Unusually Large      51.41 Why ?
PLAT143_ALERT_4_G s.u. on c - Axis Small or Missing ..............    0.00010 Ang.
PLAT242_ALERT_2_G Low    'MainMol' Ueq as Compared to Neighbors of       C00D Check
PLAT242_ALERT_2_G Low    'MainMol' Ueq as Compared to Neighbors of       C00G Check
PLAT720_ALERT_4_G Number of Unusual/Non-Standard Labels ..........         23 Note
              Ag01    F002    N003    F004    N005    O006    F007    C008
              H008    C009    C00A    C00B    H00B    C00C    C00D    F00E
              F00F    C00G    C00I    H00A    H00C    H00D    F00K
PLAT883_ALERT_1_G Absent Datum for _atom_sites_solution_primary ..     Please Do !
PLAT910_ALERT_3_G Missing FCF Reflection(s) Below Theta(Min)[Deg]=       7.26 Note
               -1  2  0,
PLAT912_ALERT_4_G Missing # of FCF Reflections Above STh/L=  0.600         18 Note
PLAT915_ALERT_3_G No Flack x Check Done: Low Friedel Pair Coverage         65 %
PLAT941_ALERT_3_G Average HKL Measurement Multiplicity ..........         4.6 Low
PLAT950_ALERT_5_G Calculated (ThMax) and CIF-Reported Hmax Differ           3 Units
PLAT951_ALERT_5_G Calculated (ThMax) and CIF-Reported Kmax Differ           3 Units
PLAT961_ALERT_5_G Dataset Contains no Negative Intensities ......     Please Check
PLAT969_ALERT_5_G The 'Henn et al.' R-Factor-gap value ..........       4.103 Note
              Predicted wR2: Based on SigI**2  2.29 or SHELX Weight  8.64
PLAT978_ALERT_2_G Number C-C Bonds with Positive Residual Density.          0 Info
```

---

0 **ALERT level A** = Most likely a serious problem - resolve or explain

```
  0 ALERT level B = A potentially serious problem, consider carefully
 10 ALERT level C = Check. Ensure it is not caused by an omission or oversight
 16 ALERT level G = General information/check it is not something unexpected

  4 ALERT type 1 CIF construction/syntax error, inconsistent or missing data
  9 ALERT type 2 Indicator that the structure model may be wrong or deficient
  6 ALERT type 3 Indicator that the structure quality may be low
  3 ALERT type 4 Improvement, methodology, query or suggestion
  4 ALERT type 5 Informative message, check
```

Validation response form

Please find below a validation response form (VRF) that can be filled in and pasted into your CIF.

```
# start Validation Reply Form
_vrf_PLAT041_lcz010
;
PROBLEM: Calc. and Reported SumFormula    Strings  Differ     Please Check
RESPONSE: ...
;
_vrf_PLAT042_lcz010
;
PROBLEM: Calc. and Reported MoietyFormula Strings  Differ     Please Check
RESPONSE: ...
;
_vrf_PLAT090_lcz010
;
PROBLEM: Poor Data / Parameter Ratio (Zmax > 18) ........       7.05 Note
RESPONSE: ...
;
_vrf_PLAT213_lcz010
;
PROBLEM: Atom F004            has ADP max/min Ratio .....        3.4 prolat
RESPONSE: ...
;
_vrf_PLAT250_lcz010
;
PROBLEM: Large U3/U1 Ratio for <U(i,j)> Tensor(Resd    1)        3.5 Note
RESPONSE: ...
;
_vrf_PLAT342_lcz010
;
PROBLEM: Low Bond Precision on  C-C Bonds ..............    0.01483 Ang.
RESPONSE: ...
;
_vrf_PLAT911_lcz010
;
PROBLEM: Missing FCF Refl Between Thmin & STh/L=    0.600          2 Report
RESPONSE: ...
;
# end Validation Reply Form
```

It is advisable to attempt to resolve as many as possible of the alerts in all categories. Often the minor alerts point to easily fixed oversights, errors and omissions in your CIF or refinement strategy, so attention to these fine details can be worthwhile. It is up to the individual to critically assess their own results and, if necessary, seek expert advice.

PLATON version of 17/09/2025; check.def file version of 17/09/2025

---

duplicate check

No duplication found

Datablock lcz010 - ellipsoid plot

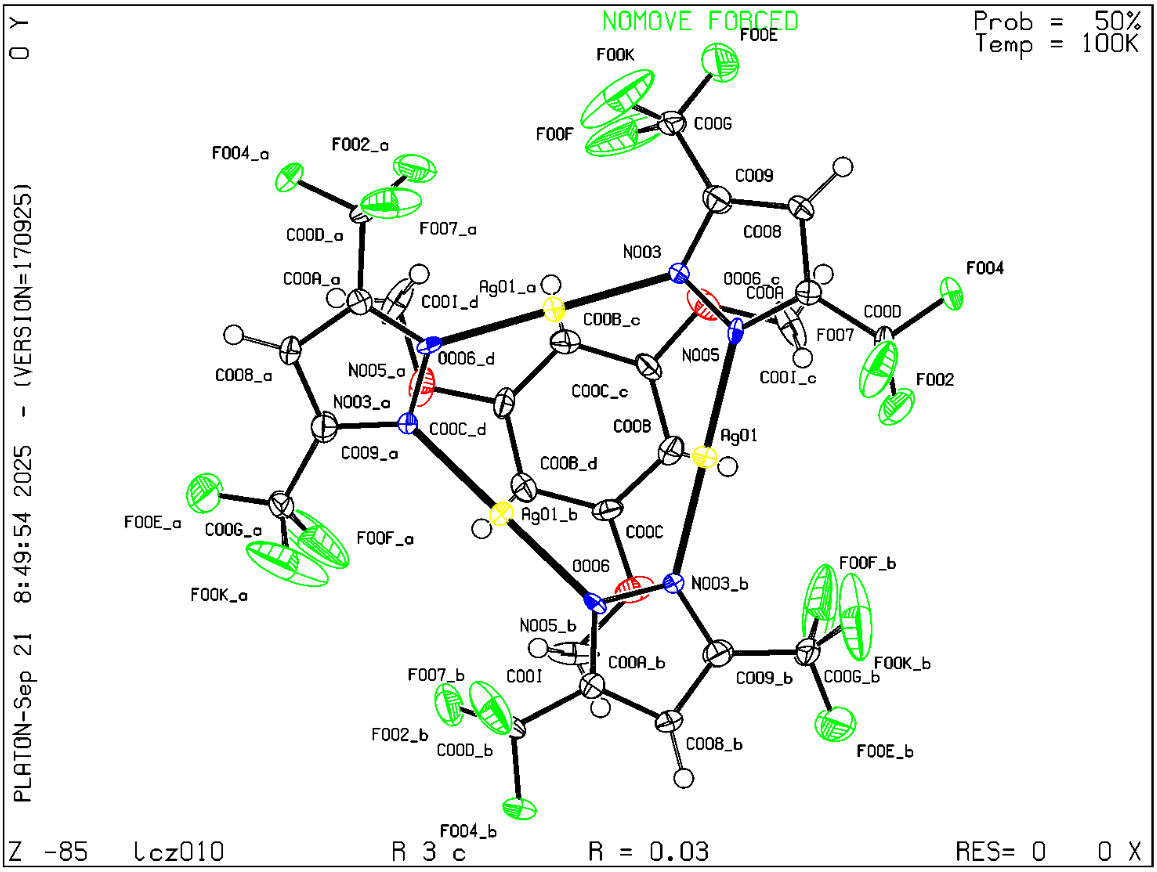

Supplement: Supplementary file 2 — Supporting File 2: anie72462‐sup‐0002‐DataFile.zip. [file ANIE-65-e1218503-s002.zip › supplementary_materials_crystallographic_data submit.zip!/cif_and_checkcif(1-114)/Ag3Pz3_10/Ag3Pz3_10_checkcif.pdf]
